# Targeted reversion of induced pluripotent stem cells from patients with human cleidocranial dysplasia improves bone regeneration in a rat calvarial bone defect model

**DOI:** 10.1186/s13287-017-0754-4

**Published:** 2018-01-22

**Authors:** Akiko Saito, Akio Ooki, Takashi Nakamura, Shoko Onodera, Kamichika Hayashi, Daigo Hasegawa, Takahito Okudaira, Katsuhito Watanabe, Hiroshi Kato, Takeshi Onda, Akira Watanabe, Kenjiro Kosaki, Ken Nishimura, Manami Ohtaka, Mahito Nakanishi, Teruo Sakamoto, Akira Yamaguchi, Kenji Sueishi, Toshifumi Azuma

**Affiliations:** 1 Department of Biochemistry, Tokyo Dental College, Tokyo, Japan; 2 Department of Orthodontics, Tokyo Dental College, Tokyo, Japan; 3 Department of Oral and Maxillofacial Surgery, Tokyo Dental College, Tokyo, Japan; 4 Oral Health Science Center, Tokyo Dental College, Tokyo, Japan; 5 Center for Medical Genetics, Keio University School of Medicine, Tokyo, Japan; 6 Laboratory of Gene Regulation, Faculty of Medicine, University of Tsukuba, Ibaraki, Japan; 7 Biotechnology Research Institute for Drug Discovery, National Institute of Advanced Industrial Science and Technology (AIST), Tsukuba, Ibaraki Japan

**Keywords:** Cleidocranial dysplasia, RUNX2, iPSCs, Osteoblasts, Osteogenesis, CRISPR/Cas

## Abstract

**Background:**

Runt-related transcription factor 2 (RUNX2) haploinsufficiency causes cleidocranial dysplasia (CCD) which is characterized by supernumerary teeth, short stature, clavicular dysplasia, and osteoporosis. At present, as a therapeutic strategy for osteoporosis, mesenchymal stem cell (MSC) transplantation therapy is performed in addition to drug therapy. However, MSC-based therapy for osteoporosis in CCD patients is difficult due to a reduction in the ability of MSCs to differentiate into osteoblasts resulting from impaired RUNX2 function. Here, we investigated whether induced pluripotent stem cells (iPSCs) properly differentiate into osteoblasts after repairing the RUNX2 mutation in iPSCs derived from CCD patients to establish normal iPSCs, and whether engraftment of osteoblasts derived from properly reverted iPSCs results in better regeneration in immunodeficient rat calvarial bone defect models.

**Methods:**

Two cases of CCD patient-derived induced pluripotent stem cells (CCD-iPSCs) were generated using retroviral vectors (OCT3/4, SOX2, KLF4, and c-MYC) or a Sendai virus SeVdp vector (KOSM302L). Reverted iPSCs were established using programmable nucleases, clustered regularly interspaced short palindromic repeats (CRISPR)/Cas-derived RNA-guided endonucleases, to correct mutations in CCD-iPSCs. The mRNA expressions of osteoblast-specific markers were analyzed using quantitative reverse-transcriptase polymerase chain reaction. iPSCs-derived osteoblasts were transplanted into rat calvarial bone defects, and bone regeneration was evaluated using microcomputed tomography analysis and histological analysis.

**Results:**

Mutation analysis showed that both contained nonsense mutations: one at the very beginning of exon 1 and the other at the initial position of the nuclear matrix-targeting signal. The osteoblasts derived from CCD-iPSCs (CCD-OBs) expressed low levels of several osteoblast differentiation markers, and transplantation of these osteoblasts into calvarial bone defects created in rats with severe combined immunodeficiency showed poor regeneration. However, reverted iPSCs improved the abnormal osteoblast differentiation which resulted in much better engraftment into the rat calvarial bone defect.

**Conclusions:**

Taken together, these results demonstrate that patient-specific iPSC technology can not only provide a useful disease model to elucidate the role of RUNX2 in osteoblastic differentiation but also raises the tantalizing prospect that reverted iPSCs might provide a practical medical treatment for CCD.

**Electronic supplementary material:**

The online version of this article (doi:10.1186/s13287-017-0754-4) contains supplementary material, which is available to authorized users.

## Background

Cleidocranial dysplasia (CCD) is a dominantly inherited disorder characterized by patent fontanelles, wide cranial sutures, hypoplasia of the clavicles, short stature, and supernumerary teeth. In addition, other skeletal anomalies and osteoporosis are common [1, 2]. Runt-related transcription factor 2 (*RUNX2*), the gene responsible for CCD [3–5], encodes a transcription factor that is a member of the runt family of proteins [6]. These proteins contain a DNA-binding domain highly homologous to the *Drosophila* pair-rule gene *runt* [7]. In particular, RUNX2 is essential for the commitment of pluripotent mesenchymal cells toward osteoblasts (OBs), the bone-forming cells [8]. Some of the mutations found in CCD have been shown to interfere with the DNA-binding activity of RUNX2*,* whereas others have been found to alter the nuclear localization of the protein or to produce a mutant or truncated protein that is biologically inactive [9–11]. *Runx*2 gene expression must be tightly regulated to support skeletal health, although sufficient activity is necessary for adequate osteogenesis because reduced or excessive *Runx2* activity may lead to osteoporosis [12]. Mice that are deficient in RUNX2 have complete absence of bone [5, 13, 14], while those with haploinsufficiency of this transcription factor mimic some of the human CCD phenotypes, while others, such as supernumerary teeth, some skeletal abnormalities, and osteoporosis, have not been clearly demonstrated in mice [13].

Osteoporosis is a debilitating disease caused by systemic bone loss in the musculoskeletal system. Other than the anabolic parathyroid hormone drug, current Food and Drug Administration (FDA)-approved treatments are predominant agents that aim to inhibit bone resorption and prevent further bone loss. These types of treatment are associated with serious side effects and consequently there is a call for an alternative approach to treat osteoporosis [15]. The number of clinical trials involving mesenchymal stem cell (MSC)-based therapy has increased markedly over the last decade. This is because MSCs are multipotent adult stem cells that can be easily procured from various sources, such as bone marrow, adipose, and cord blood tissue [16].

However, in osteoporosis, the number of bone marrow MSCs that can differentiate into osteoblasts and form bone is significantly reduced. In addition, MSC derived from CCD patients seems to have a decreased ability to induce differentiation into osteoblasts due to haploinsufficiency of RUNX2.

Human induced pluripotent stem cells (hiPSCs) provide an iPSC-based novel disease model as well as a therapeutic option for the treatment of CCD [17]. We previously reported an efficient method to differentiate hiPSCs into osteogenic lineage cells [18] that supported the notion to generate CCD patient-derived iPSCs to elucidate the pathophysiology of CCD.

However, patient-derived iPSCs contain genetic mutations and exhibit poor osteogenic differentiation capability. Furthermore, hiPSCs have different genetic backgrounds and could exhibit heterogeneity, thereby limiting their use as model systems [19]. A technique has been developed to correct the defective gene in patient-derived iPSCs using programmable nucleases, i.e., clustered regularly interspaced short palindromic repeats (CRISPR)/Cas-derived RNA-guided endonucleases, which is now widely used for genomic editing of higher eukaryotic cells [20]. In this RNA-guided nuclease system, a user-defined single-guide RNA (sgRNA) directs the endonuclease Cas9 to a specific genomic target, where it cleaves chromosomal DNA. This cleavage activates endogenous cellular DNA repair pathways in a process known as homologous recombination, which gives rise to targeted correction of mutated genes [21]. In this report, we show the successful correction of the mutated RUNX2 gene, and show that this genetic correction gives rise to normal osteogenic potential in patient-derived iPSCs. Our evidence clearly reveals that reverted iPSCs could be used as a source for regeneration therapy.

Herein, we report the generation of two types of CCD patient-derived iPSCs, one with a heterozygous loss of DNA-binding activity of RUNX2 and another which supposedly alters nuclear localization of the RUNX2 protein. Both types showed impaired osteodifferentiation capabilities. We further generated reverted iPSCs to restore the proper osteodifferentiation potential not only in vitro but also in vivo. These iPSCs should prove valuable for elucidating disease mechanisms and providing an iPSC-based novel therapeutic option for the treatment of CCD or other diseases related to the Runx2 gene.

## Methods

### Cell culture

All experimental procedures described in this manuscript were approved by the Ethics Committee of Tokyo Dental College (permit no. 533). Primary human oral fibroblasts (OFs) were prepared from oral mucosal tissue (5 × 10 mm) resected from CCD patients, which were then cultured and subjected to reprogramming by infecting with retroviral vectors (OCT3/4, SOX2, KLF4, and c-MYC) or a Sendai virus SeVdp vector (KOSM302L) as described previously [22–24]. All iPSCs were maintained in human embryonic stem cell (ESC) media (Dulbecco’s modified Eagle’s medium/nutrient mixture F-12 + GlutaMAX media supplemented with 20% knockout serum replacement media, 1% l-glutamine, 1% nonessential amino acids, 1% penicillin–streptomycin, 0.1 mM 2-mercaptoethanol, and 5 ng/ml fibroblast growth factor (FGF)-2).

The efficient method of differentiating hiPSCs into osteogenic lineage cells is shown in our previous study [18] as well as in Fig. 2a. For osteoblast differentiation, cells were cultured in osteoblast differentiation medium (OBM), which consisted of alpha-MEM supplemented with 10% fetal bovine serum (FBS), 50 mg/ml l-ascorbic acid, 10 mM β-glycerophosphate, and 10 nM dexamethasone, supplemented with cytokines (25 ng/ml FGF-2, 1 ng/ml transforming growth factor (TGF)-β1, 100 ng/ml insulin-like growth factor (IGF)-1) for 12 days. OBM containing fresh cytokines was resupplied every 3 days.

Primary human osteoblasts (HOBs) derived from normal bone tissue were purchased from Takara Bio Inc. (Shiga, Japan) and cultured in osteoblast growth medium (Takara Bio Inc.).

### Mutation analysis

Exons 1–7 of the *RUNX2* gene were amplified by polymerase chain reaction (PCR) under standard conditions for analyzing CCD case 1 (CCD1) and CCD case 2 (CCD2). The primers used for genomic PCR amplification and sequencing are described elsewhere [25].

### CRISPR/sgRNA design and construction

**Fig. 1:**
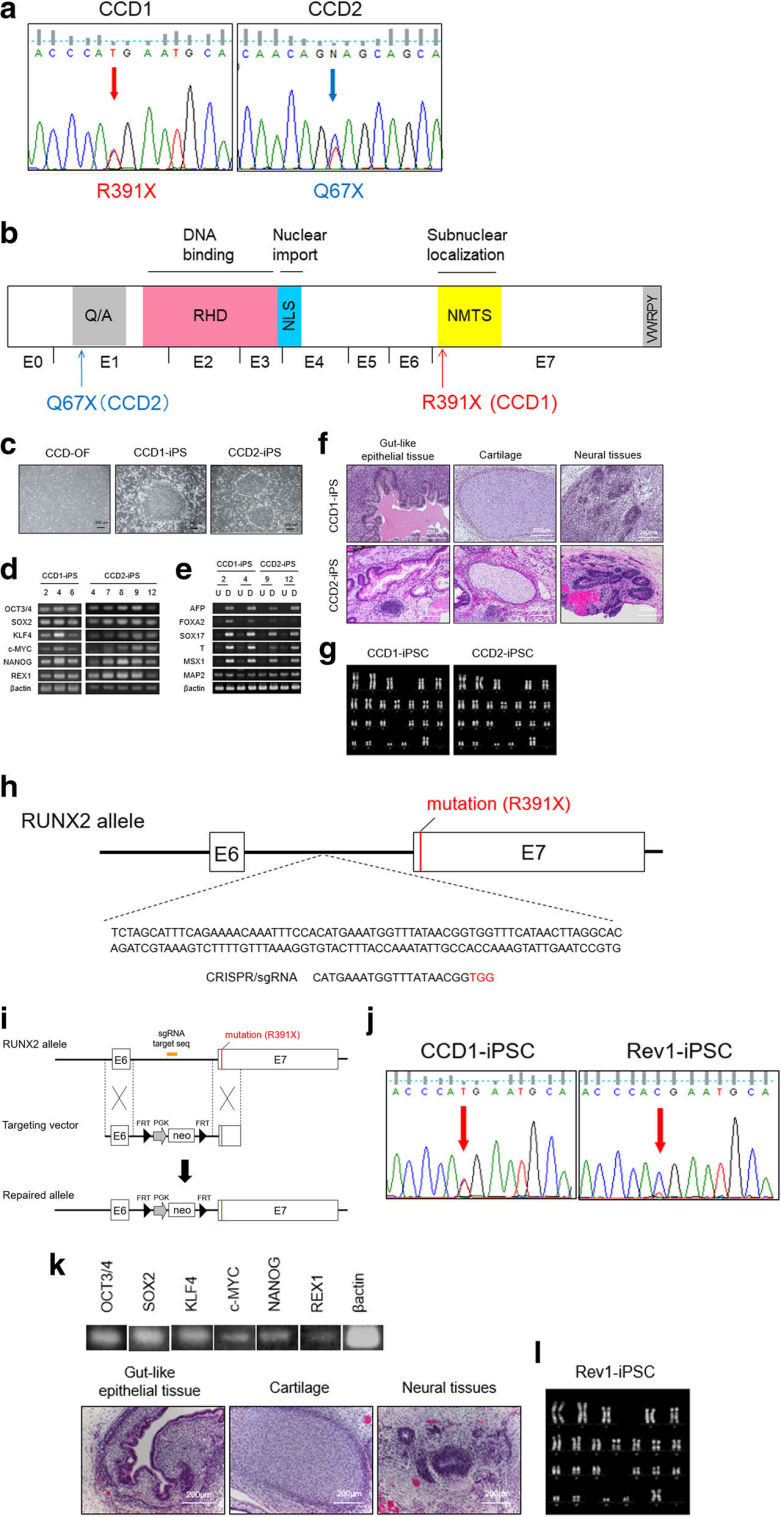
Generation of cleidocranial dysplasia (CCD) patient-specific iPSCs and genome editing. **a** Identification of heterozygous runt-related transcription factor 2 (*RUNX2*) mutations in each CCD. **b** Functional domains of RUNX2 proteins and RUNX2 exon organization. **c** Morphology of established CCD patient-derived induced pluripotent stem cells (CCD-iPS). **d–f** Confirmation of the pluripotency of the CCD-iPSCs. **d** RT-PCR analysis of ESC marker genes. **e** RT-PCR analyses of various differentiation markers for the three germ layers. Target genes included *AFP*, *FOXA2*, and *SOX17* (endoderm), *T* and *MSX1* (mesoderm), and *MAPs* (ectoderm). *β-actin* was used as an internal control. **f** Teratoma formation and histology. Teratoma contained tissues of three embryonic germ layers: cartilage (mesoderm), gut-like epithelium tissues (endoderm), and neural tube-like structures (ectoderm). **g** Karyotype analysis (Q-band) of CCD1- and CCD2-iPSCs. **h** Clustered regularly interspaced short palindromic repeats (CRISPR)/single-guide RNA (sgRNA) targeting of the *RUNX2* gene on chromosome 6. **i** CRISPR-mediated genome editing of CCD1-iPSCs. **j** Confirmation of the *RUNX2* gene correction by sequence analysis. **k** Confirmation of the pluripotency of the Reverted iPSCs (Rev1-iPSCs). **l** Karyotype analysis (Q-band) of Rev1-iPSCs. Abbreviations: D, differentiated; U, undifferentiated; Q/A, glutamine/alanine-rich domain; RHD, runt homology domain; NLS, nuclear localization signal; NMTS, nuclear matrix-targeting signal; FRT, flippase recognition target; PGK, phosphoglycerate kinase

We explored the sgRNA using the CRISPRdirect bioinformatics tool against the human RUNX2 locus located on chromosome 6. Complete sequences of the target sites of the sgRNA are provided in Fig. 1g. The targeting donor vector was constructed based on a DT-A/conditional knockout FW plasmid (RIKEN Center for Developmental Biology, Kobe, Japan) with several modifications. The PCR-amplified fragments from genomic DNA of a healthy individual were inserted into the restriction enzyme-digested plasmid.

### CRISPR/sgRNA transfections for reversion in iPSCs

Transfection of the CRISPR/sgRNA plasmid into iPSCs was performed as described previously [26]. Electroporation was performed using a Neon Transfection System (Thermo Fisher Scientific, Waltham, MA, USA) according to the manufacturer’s instructions. Briefly, iPSCs were harvested by treating with 0.05% trypsin-ethylenediaminetetraacetic acid (EDTA) solution (Thermo Fisher Scientific). Cells (1 × 10^6^) were suspended in 100 μl electroporation buffer containing 5 μg of the donor plasmid and 5 μg of the CRISPR/sgRNA expression plasmid, and electroporated (condition: 1050 V, 20 ms, two pulses). After electroporation, the cells were subsequently plated on an iMatrix-511 (Nippi Inc., Tokyo, Japan)-coated 100-mm dish and cultured in StemFit® AK01 medium (Ajinomoto, Tokyo, Japan) supplemented with ROCK inhibitor (Y-27632; Wako Pure Chemical Industries Ltd.) for the first 24 h. Geneticin selection (300 μg/ml) was started 72 h after electroporation. Individual colonies were picked and expanded for 10 days after electroporation.

### RNA isolation, reverse transcriptase PCR, and quantitative PCR

Total RNA was extracted using QIAzol reagent (Qiagen, Hilden, Germany) according to the manufacturer’s instructions. Complementary DNA (cDNA) was synthesized using a high-capacity cDNA reverse transcription kit (Thermo Fisher Scientific). To confirm the expression of markers of ESCs and three germ layers, PCR was performed with Ex-Taq polymerase (Takara Bio, Inc.). β-actin was used as an internal control. All primer sets are described elsewhere [24]. Quantitative reverse transcriptase (qRT)-PCR was performed using Premix Ex-Taq reagent (Takara Bio, Inc.) according to the manufacturer’s instructions. 18S rRNA was used as an internal control. All primer sequences are shown in Table 1. The relative expression of genes of interest was estimated using the ΔΔCt method.

**Table 1:** Primers used for quantitative reverse transcriptase polymerase chain reaction

Gene symbol	GenBank accession no.	Forward primer sequence	Reverse primer sequence
RUNX2	NM_001024630.2	gtgcctaggcgcatttca	gctcttcttactgagagtggaagg
ALP	NM_000478.3	caaccctggggaggagac	gcattggtgttgtacgtcttg
COL1A1	NM_000088.3	gggattccctggacctaaag	gggattccctggacctaaag
OSX	NM_152860.1	catctgcctggctccttg	caggggactggagccata
OC	NM_199173.3	tgagagccctcacactcctc	acctttgctggactctgcac
MSX2	NM_002449.4	tcggaaaattcagaagatgga	caggtggtagggctcatatgtc
DLX5	NM_005221.5	ctacaaccgcgtcccaag	gccattcaccattctcacct
DLX3	NM_005220.2	gagcctcctaccggcaatac	tcctccttcaccgacactg
TWIST1	NM_000474.3	agctacgccttctcggtct	ccttctctggaaacaatgacatc
18SrRNA	M11188.1	cggacaggattgacagattg	cgctccaccaactaagaacg

### Immunofluorescent staining

Cells cultured on cover glasses were fixed in 4% paraformaldehyde in phosphate-buffered saline (PBS) for 15 min, permeabilized with ice-cold 100% methanol for 10 min at −20 °C, and then blocked with 5% normal goat serum/0.3% Triton X-100 in PBS for 1 h. Subsequently, the cells were incubated with anti-RUNX2 antibody (dilution 1:1600; Cell Signaling Technology, Inc., Beverly, MA, USA), followed by Alexa Fluor® 488-conjugated goat anti-rabbit or mouse immunoglobulin G. Localization of the RUNX2 protein was visualized by immunofluorescent microscopy (UPM AxiophoT2; Carl Zeiss Microscopy, LLC, Thornwood, NY, USA).

### Preparation of scaffold/iPSC-derived OB complex for transplantation

Osteogenic differentiation from iPSCs was performed according to Fig. 3a. After 4 weeks, the iPSC-derived OBs were dissociated with 0.5 mg/ml collagenase type IV for 30 min and 0.25% trypsin-EDTA for 5 min at 37 °C. To facilitate the self-assembly of a peptide nanofiber scaffold-derived three-dimensional (3D) culture, we used PuraMatrix (BD Biosciences, Cambridge, MA, USA) to encapsulate the cells; 10 μl of freshly dissociated cells (2 × 10^5^) suspended in serum-free media was mixed with 10 μl of the 2.5% PuraMatrix. Gelation was initiated after the peptide solution was mixed with the cell suspension, thereby resulting in cell encapsulation inside the nanofiber hydrogel.

### Transplantation of scaffold/iPSC-derived OB complex

All procedures performed with live animals conformed to the ethical guidelines established by the Japanese Council on Animal Care and were approved by the Animal Care Committee of Tokyo Dental College (permit no. 290401). Seven-week-old male F344/NJcl-rnu/rnu rats were obtained from Clea Japan, Inc. (Tokyo, Japan). After anesthesia induction with 4% sevoflurane (Maruishi Pharmaceutical Co. Ltd., Osaka, Japan) inhalation, the rats were further anesthetized by intraperitoneal injection with sodium pentobarbital (30 mg/kg body weight; somnopentyl; Kyoritsu Seiyaku, Tokyo, Japan). Rat calvarial bone defects were created as previously described [27]. Scaffold/iPSC-derived OB complex was injected into the bone defect. The rats receiving transplantation were sacrificed at 4 weeks and subjected to radiographical and histological analyses.

### Radiographical analyses and histological assessment

Microcomputed tomography (CT) parameters were as follows: X-ray source, 90 kV/100 μA; rotation, 360°; exposure time, 17 s; and voxel size, 50 × 50 × 50 μm (R-μCT®; Rigaku Corporation, Tokyo, Japan). CT images were compiled and three-dimensional images were rendered using the TRI/3D-BON system (Ratoc System Engineering Co. Ltd., Tokyo, Japan) as described previously [27]. Coronal sections (thickness = 5 μm) through the center of each circular defect were prepared and Villanueva–Goldner (V–G) staining [28] was performed.

### Statistical analysis

All data are expressed as the mean ± standard deviation (SD). When analysis of variance indicated differences among the groups, multiple comparisons among the experimental groups were performed using the Bonferroni test. Statistical significance was defined as *p* < 0.05.

## Results

Sequencing analysis of the genomic DNA extracted from the CCD-OFs revealed a heterozygous nonsense mutation (R391X as CCD1, Q67X as CCD2) in the *RUNX2* gene (Fig. 1a). This mutation corresponds with the functional domain of the RUNX2 protein, nuclear matrix-targeting signal (NMTS) (CCD1), and the glutamine/alanine-rich domain (Q/A) (CCD2) (Fig. 1b). More than three iPSC lines were established and analyzed for each CCD patient. Patient-specific iPSCs from CCD-OFs (Fig. 1c) were generated using retroviral or Sendai viral vectors that encode the four Yamanaka factors [23, 24]. All the retroviral or Sendai viral iPSC lines suppressed transgenes (data not shown). The pluripotency of the CCD-iPSC lines was confirmed as shown in Fig. 1d–f. CCD-iPSCs displayed basic properties of pluripotent cells, as indicated by the expression of the embryonic stem cell markers *OCT3/4*, *NANOG*, and *REX1* (Fig. 1d), the expression of the three germ-layer differentiation markers in embryoid bodies (Fig. 1e), the ability to form teratomas (Fig. 1f), and a normal karyotype (Fig. 1g). Reverted iPSC (Rev1-iPSC) lines were generated using CRISPR/Cas9-derived RNA-guided endonucleases (Fig. 1h, i). RUNX2 gene correction was confirmed by sequence analysis (Fig. 1j). Rev1-iPSCs were maintained in a pluripotent state, as indicated by the expression of the pluripotency markers, and displayed the ability to form teratomas (Fig. 1k), and a normal karyotype (Fig. 1l).

**Fig. 2:**
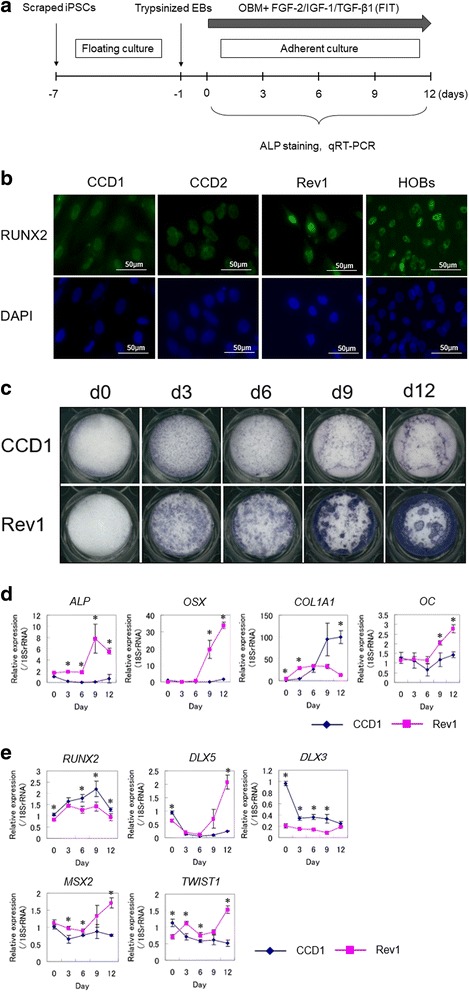
Osteoblastic differentiation of induced pluripotent stem cells (iPSCs) in vitro. **a** Schedule of osteogenic differentiation. **b** Runt-related transcription factor 2 (RUNX2) localization by immunofluorescent microscopy. Single cells from embryoid bodies (EBs) were cultured with osteoblast differentiation medium (OBM) containing cytokines for 12 days and stained for RUNX2 (green) and the nuclei (blue). **c** Staining of alkaline phosphatase (ALP) activity (days (d)0, 3, 6, 9, and 12). **d** qRT-PCR analysis of RUNX2 target genes. **e** qRT-PCR analysis of *RUNX2* and transcription factors. Values are presented as the mean ± SD (*n* = 3). *p < 0.05. FGF fibroblast growth factor, HOB human osteoblast, IGF insulin-like growth factor, TGF transforming growth factor

Osteogenic differentiation from iPSCs was performed according to our previous report [18] and Fig. 2a. CCD-OBs showed a decrease in the nuclear localization of RUNX2 (Fig. 2b). However, RUNX2 localization was restored in Rev1-OBs to the same level as normal primary HOBs (Fig. 2b). Furthermore, we examined Runx2 localization in calvarial OBs derived from Runx2 wild-type (Runx2^+/+^) and heterozygous knock-out (Runx2^+/–^) mice. As a result, Runx2 localization was decreased in Runx2^+/–^-OBs compared to Runx2^+/+^-OBs (Additional file 1: Figure S1). We determined the expression of OB-specific markers to confirm OB differentiation. As expected, in OBM with FGF-2, TGF-β1, and IGF-1 greatly increased the alkaline phosphatase (ALP) activity of Rev1-iPSCs, but not CCD1-iPSC, in a time-dependent manner (Fig. 2c). Furthermore, we examined ALP activity of primary HOBs and mouse OBs (mOBs) as controls. As a result, the ALP activity of Rev1-iPSCs cultured in OBM for 12 days was at the same level as HOBs and Runx2^+/+^-mOBs. On the other hand, the ALP activity of Runx2^+/–^-mOBs was decreased similarly to CCD1-iPSCs (Additional file 2: Figure S2). As compared with that in CCD1-iPSCs, the expression levels of the OB-specific markers *ALP*, *OSX*, and *OC* were markedly upregulated only in Rev1-iPSCs cultured in OBM for 9 days (Fig. 2d). The mRNA expression level of *RUNX2* was higher in CCD1-iPSCs than in Rev1-iPSCs (Fig. 2e). We next investigated major homeodomain (HD) proteins in osteogenesis, such as *DLX5*, *DLX3, MSX2*, and *TWIST1* [29]. The expression levels of *DLX5*, *MSX2*, and *TWIST1* mRNA molecules were sharply increased in Rev1-iPSCs cultured in OBM after 9 days, but not in CCD1-iPSCs (Fig. 2e). The expression level of *DLX3* was higher in CCD1-iPSCs than in Rev1-iPSCs (Fig. 2e). These observations indicated that the osteolineage differentiation of CCD1-iPSCs was significantly delayed, relative to that of Rev1-iPSCs.

**Fig. 3:**
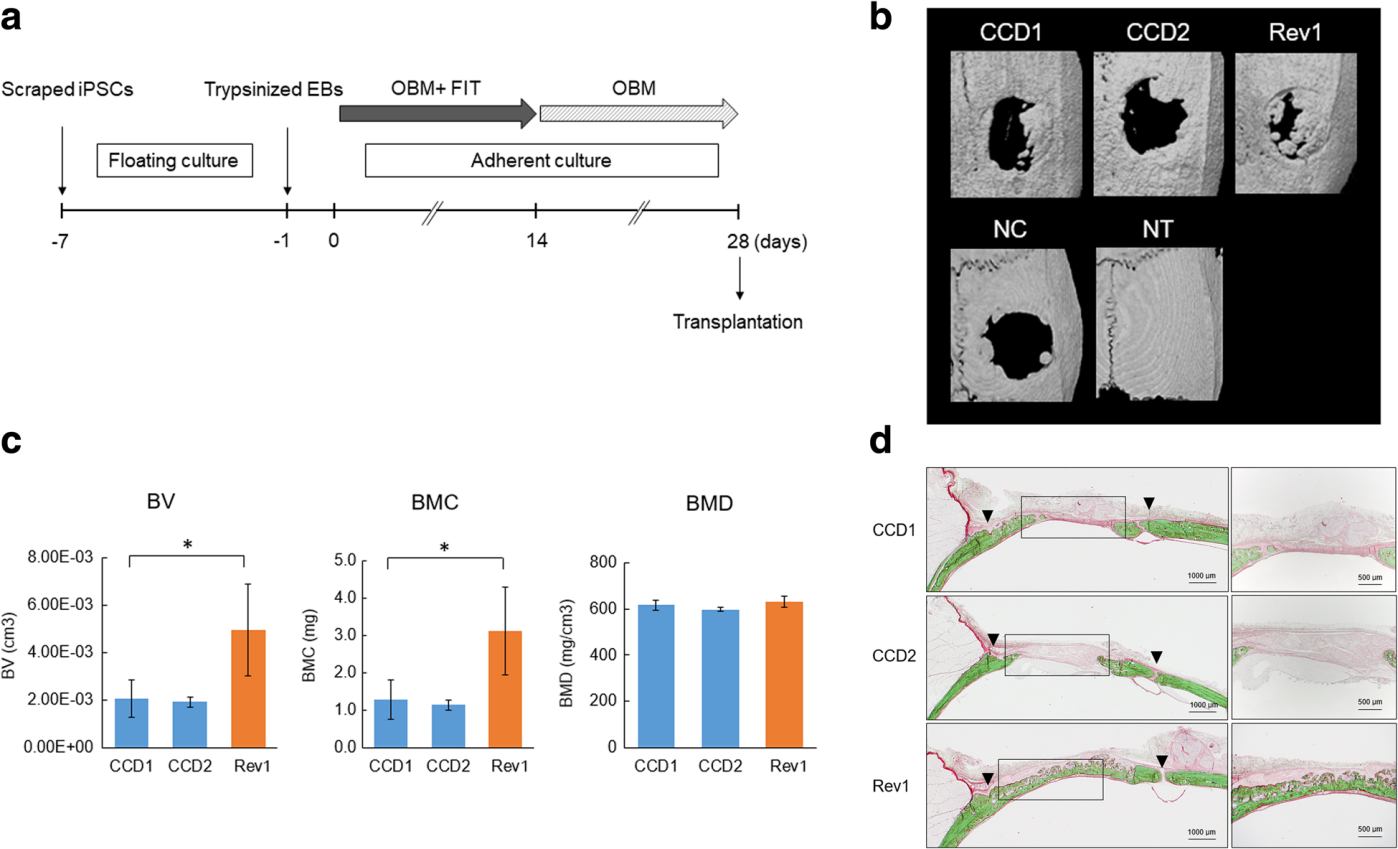
Transplantation of induced pluripotent stem cell (iPSC)-induced osteoblasts (OBs) and bone regeneration. **a** Transplantation protocol for rat calvarial bone defects. **b** MicroCT images of the calvaria at 4 weeks after transplantation in the CCD1, CCD2, and Rev1 groups (*n* = 3). **c** MicroCT analysis of bone regeneration. Comparison of new bone volume (cm^3^) in the regions of interest of three groups. Values are presented as the mean ± SD (*n* = 3). *p < 0.05. **d** V–G staining images at 4 weeks after transplantation. Top: CCD1 group. Middle: CCD2 group. Lower: Rev1 group. Magnified images of the new bone area are shown on the right. BMC bone mineral content, BMD bone mineral density, BV bone volume, EB embryoid body, NC nontransplanted defect (negative control), FIT fibroblast growth factor/insulin-like growth factor/transforming growth factor, NT nontreated control, OBM osteoblast differentiation medium

MicroCT images of the calvaria at 4 weeks after transplantation are shown in Fig. 3b. Re-ossification developed via growth extension from the bony rims at the lateral edges of the bone defects. Newly generated bone was observed 4 weeks after surgery in the Rev group. Minimal new bone was observed in the two CCD groups (Fig. 3b). The bone volume (BV) and bone mineral content (BMC) were significantly higher in the Rev group than in the CCD groups (Fig. 3c). On the other hand, there was no difference in bone mineral density (BMD) (Fig. 3c). Histological examination by V–G staining provides uniform and reproducible results with mineralized or undecalcified bone. Mineralized bone tissues are stained green and nonmineralized osteoid tissues are stained red by V–G stain (Fig. 3d). Newly formed bone was observed around the margin and along the intracranial periosteum, as well as in the center of the bone defects, including around the top portion of the defect area in the Rev group. In the CCD groups, there was comparatively less newly formed bone (Fig. 3d), indicating that CCD-iPSCs may have delayed osteodifferentiation.

## Discussion

In this study we present two major results. First, we successfully corrected the mutated Runx2 gene in iPSCs using the CRISPR/Cas method. Second, the reverted iPSCs that we generated exhibited better osteogenic properties both in vivo and in vitro, suggesting that these iPSCs provide not only an ideal source for studying CCD but also provide an iPSC-based novel therapeutic option for the treatment of CCD or other diseases related to the RUNX2 gene.

We showed that CCD-iPSCs were found to have poor osteogenic differentiation capability, as the expression levels of transcription factors important for osteodifferentiation (i.e., HD protein, *OSX*, *TWIST1*, *MSX2*, and *DLX5*) were not increased in the proper manner observed in Rev-iPSCs cultured in OBM after 9 days. An in vivo experiment (calvarial defects model) revealed the poor regeneration capability of CCD-OBs relative to that of Rev-OBs.

In the present study, two types of CCD-iPSC derived from patients enrolled in this study were generated and each had a stop codon insertion mutation in the Q/A-rich domain or NMTS domain. Nonsense mutation in the Q/A-rich domain resulted in complete loss of runt domain, which is a responsive sequence for binding to Runx2 target cic-element. On the other hand, NMTS is an important domain for nuclear localization [30]. Deficiency of this domain significantly impeded nuclear localization of RUNX2 and reduced the coordinated action of the Smad signaling [30, 31]. These Runx2 functions were some of the most important functional domains in the Runx2 gene. The deficiency of any domain similar to a loss of function of the transcription factor of RUNX2 has been reported to result in failure of bone and cartilage differentiation [30–33]. Similarly, we found that the osteodifferentiation capability of the two cases of CCD-iPSCs we examined were poor.

We showed that Rev-iPSCs, which could have restored the proper osteodifferentiation potential, were generated by the CRISPR/Cas method. We sequenced and confirmed the corrected mutated sequence. These Rev-iPSCs still maintained pluripotencies but gained better osteogenic abilities. iPSCs provide an attractive platform for the study of rare diseases, including CCD. Patient-derived iPSCs have great potential as a disease model because they can generate unlimited quantities of cells. Furthermore, genetic manipulations, such as the introduction of exogenous genes and specific correction of a defined mutation in established iPSCs, could lead to novel cellular therapies. To date, there have been few reports of successfully generated corrected iPSCs (Rev-iPSCs) from patients [34–37]. Another important reason to generate Rev-iPSCs is their utility as a control for patient-derived iPSCs in research. Therefore, we established Rev-iPSCs as control cells from these CCD-iPSCs using the CRISPR/Cas tool.

RUNX2–HD protein complexes regulate target genes, and RUNX2 has been characterized as a major hub of HD protein regulation [29, 38–41]. Hojo et al. also reported that osterix (Osx) is restricted to bone-forming vertebrates, where it acts as a Dlx co-factor in OB specification [39]. We investigated major HD proteins in osteogenesis from human-derived iPSCs and found that insufficiency of RUNX2 might disrupt the early osteogenic circuitry for commitment of the bone cell phenotype in the differentiation from iPSC to osteolineage cells. Recent reports also described that aged mice required full *Runx2* gene dosage for cancellous bone regeneration after bone marrow ablation [42]. Experiments with a calvarial bone defect model using severe combined immunodeficiency rats to transplant CCD-OBs showed poor bone formation, while reverted CCD-OBs showed substantially improved bone regeneration. However, there was no significant difference in BMD among all three groups. Thus, RUNX2 may have a weak effect on BMD owing to its regulation, which covers a relatively early stage, but not late stage, of osteoblast differentiation. These findings suggest that the haploinsufficiency of RUNX2 has a significant effect on the early stage of membrane ossification, which causes clinical symptoms in patients with CCD, and that reverted iPSCs provide an iPSC-based novel therapeutic option for the treatment of CCD.

## Conclusions

iPSCs were generated from patients with CCD, and Rev-iPSCs with corrected mutations were created by CRISPR/Cas-mediated genome editing. These iPSCs can not only provide a useful disease model to elucidate the role of RUNX2 in osteoblastic differentiation but also raise the tantalizing prospect that reverted iPSCs might provide a practical medical treatment for CCD.

## Additional files

Additional file 1: Figure S1. RUNX2 localization in primary calvarial osteoblasts (OBs) derived from Runx2 wild-type mice (Runx2^+/+^ mOBs) and Runx2 heterozygous knock-out mice (Runx2^+/–^ mOBs) by immunofluorescent microscopy. RUNX2 (green) and the nuclei (blue). (TIFF 237 kb)

Additional file 2: Figure S2. Staining of ALP activity in HOBs and mOBs (Runx2^+/+^ and Runx2^+/–^). (TIFF 350 kb)

## Abbreviations

ALP: Alkaline phosphatase
BMC: Bone mineral content
BMD: Bone mineral density
BV: Bone volume
CCD: Cleidocranial dysplasia
cDNA: Complementary DNA
CRISPR: clustered regularly interspaced short palindromic repeats
CT: Computed tomography
EDTA: Ethylenediaminetetraacetic acid
ESC: Embryonic stem cell
FGF: Fibroblast growth factor
hiPSC: Human induced pluripotent stem cell
HOB: Human osteoblast
IGF: Insulin-like growth factor
iPSC: Induced pluripotent stem cell
mOB: Mouse osteoblast
MSC: Mesenchymal stem cell
NMTS: Nuclear matrix-targeting signal
OB: Osteoblast
OBM: Osteoblast differentiation medium
OF: Oral fibroblast
OSX: Osterix
PBS: Phosphate-buffered saline
PCR: Polymerase chain reaction
qRT-PCR: Quantitative reverse transcriptase polymerase chain reaction
RUNX2: Runt-related transcription factor 2
SD: Standard deviation
sgRNA: Single-guide RNA
TGF: Transforming growth factor
V–G: Villanueva–Goldner
