# Supplementary figures and images for: Targeted reversion of induced pluripotent stem cells from patients with human cleidocranial dysplasia improves bone regeneration in a rat calvarial bone defect model
Source: Stem Cell Res Ther. 2018 Jan 22;9:12. doi: 10.1186/s13287-017-0754-4 (PMC5778688; doi:10.1186/s13287-017-0754-4)

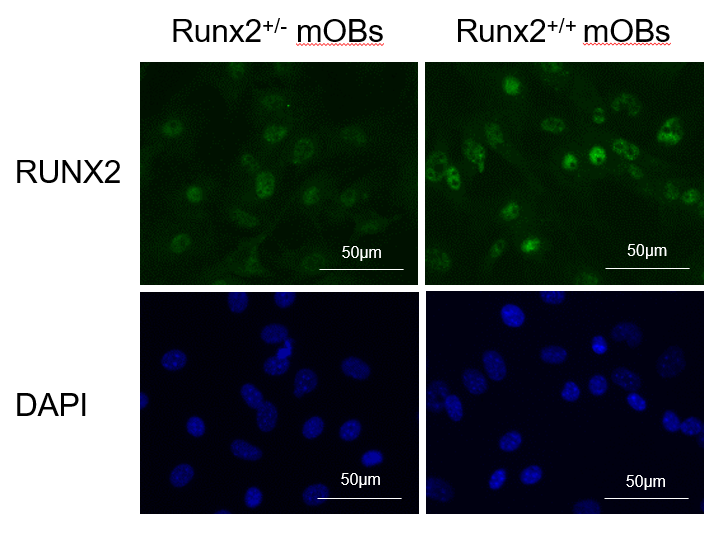

Supplement: Supplementary file 1 — RUNX2 localization in primary calvarial osteoblasts (OBs) derived from Runx2 wild-type mice (Runx2+/+ mOBs) and Runx2 heterozygous knock-out mice (Runx2+/– mOBs) by immunofluorescent microscopy. RUNX2 (green) and the nuclei (blue). (TIFF 237 kb) [file 13287_2017_754_MOESM1_ESM.tiff]

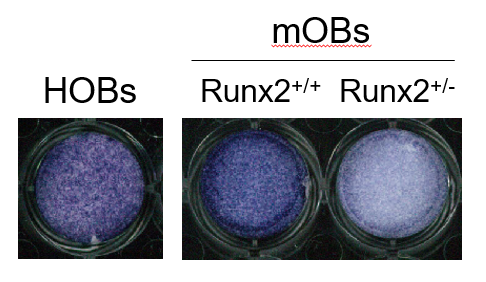

Supplement: Supplementary file 2 — Staining of ALP activity in HOBs and mOBs (Runx2+/+ and Runx2+/–). (TIFF 350 kb) [file 13287_2017_754_MOESM2_ESM.tiff]
